# How Might People Near National Roads Be Affected by Traffic Noise as Electric Vehicles Increase in Number? A Laboratory Study of Subjective Evaluations of Environmental Noise

**DOI:** 10.1371/journal.pone.0150516

**Published:** 2016-03-03

**Authors:** Ian Walker, John Kennedy, Susanna Martin, Henry Rice

**Affiliations:** 1 Department of Psychology, University of Bath, Bath, United Kingdom; 2 Department of Mechanical Engineering, Trinity College Dublin, Dublin, Ireland; Beihang University, CHINA

## Abstract

We face a likely shift to electric vehicles (EVs) but the environmental and human consequences of this are not yet well understood. Simulated auditory traffic scenes were synthesized from recordings of real conventional and EVs. These sounded similar to what might be heard by a person near a major national road. Versions of the simulation had 0%, 20%, 40%, 60%, 80% and 100% EVs. Participants heard the auditory scenes in random order, rating each on five perceptual dimensions such as pleasant–unpleasant and relaxing–stressful. Ratings of traffic noise were, overall, towards the negative end of these scales, but improved significantly when there were high proportions of EVs in the traffic mix, particularly when there were 80% or 100% EVs. This suggests a shift towards a high proportion of EVs is likely to improve the subjective experiences of people exposed to traffic noise from major roads. The effects were not a simple result of EVs being quieter: ratings of bandpass-filtered versions of the recordings suggested that people’s perceptions of traffic noise were specifically influenced by energy in the 500–2000 Hz band. Engineering countermeasures to reduce noise in this band might be effective for improving the subjective experience of people living or working near major roads, even for conventional vehicles; energy in the 0–100 Hz band was particularly associated with people identifying sound as ‘quiet’ and, again, this might feed into engineering to reduce the impact of traffic noise on people.

## Introduction

Motor vehicles produce noise as a by-product of their operation, mostly from engine sound and tyre-road contact. Vehicle noise can be considered from various perspectives–for example, there is a body of work on how sound might be used by pedestrians to detect the movements of vehicles in urban spaces [1,2]. Our interest here lies with the subjective experience of traffic noise for people who live or work near major roads.

Previous research suggests the presence of traffic noise from such roads is associated with detrimental effects on sleep quality [3], mental health [4] and overall quality of life [5,6]. The World Health Organization [7] estimated that Europe sees around 1.7 million Disability Adjusted Life Years (DALYs) lost each year as a result of environmental noise–of which traffic is a key component. Traffic noise has also been implicated in impairments to children’s cognitive function at school [8] and conduct problems [9]. At a lower level, studies have associated exposure to traffic noise with harmful physiological outcomes such as hypertension and cardiovascular disease [10]. Overall, the literature paints a clear picture in which traffic noise places a considerable burden on society. However, more work is needed to understand the processes by which vehicle noise affects those exposed to it [11].

Against this background, there is currently considerable interest in moving from conventional oil-powered vehicles towards electric vehicles (EVs) and hybrid vehicles over the next couple of decades. EVs have the potential to show both qualitative and quantitative changes in the sound they produce–they might sound different and also be quieter overall [12–19]. This study asked what effect the adoption of EVs might have on the subjective experience of people who hear them: is a shift towards more EVs likely to increase or decrease the aforementioned negative effects on people? The study was part of a larger international project about national roads called FOREVER (Future OpeRational impacts of Electric Vehicles on National European Roads–see [20]), and so we concentrate exclusively on this situation here. Focusing on the case of national roads also means this study complements and extends the small existing body of work on EV noise, which has tended so far to focus on urban settings.

Clearly, using real roads to investigate a hypothetical change in the make-up of a nation’s vehicle fleet is impossible, and so this study was carried out in the laboratory. The procedure asked participants to listen to various *auralizations* (synthesized spatialized stereo recordings) of traffic noise over headphones. These reproduced the auditory experience of a person near a busy national road. Participants rated each instance on several perceptual dimensions, such as pleasant-unpleasant and relaxing-stressful. The soundscapes were synthesized using information from an earlier analysis of real conventional and electric cars driving past a microphone array in controlled conditions (Kennedy, Rice & Walker, in prep), based in turn on a procedure used by Smyth, Rice, McDonald and Gerdelan [21]. Using genuine pass-by recordings ensured that the auralizations included the effects of directivity, frequency content and level recorded in accordance with ISO standard measurement procedures, and no incidental sounds that might influence participants’ judgements.

The auralizations included conditions with 100% conventional vehicles and 100% EVs, as well as various intermediate mixtures. Asking participants to rate their experience of these sounds using several perceptual scales was based on the procedure of Giudice, Jennings, Cain, Humphreys and Song [22]. We preferred this approach to the simple annoyance ratings often used in this area (e.g., [23,24]) as such measures are not only unidimensional, but also assume (and communicate to participants) that subjective experience will be unpleasant, making the participant’s task simply to assess *how* negative. Our approach was more open and did not communicate any expectation to participants that the sounds would be unpleasant.

Each auralization was judged in isolation from the others, with their order randomized for each participant; the procedure involved no side-by-side comparison between auralizations. This removes a concern that might be found with, say, a two-alternative forced-choice paradigm in which participants had to select the most (un)pleasant sound. A method like that obliges participants to class one stimulus as being higher than the other on the perceptual dimension, even if they cannot tell the two apart. The procedure used here potentially allows participants to rate all stimuli the same if they cannot tell any difference, meaning that if any differences in subjective experience do emerge, we can interpret these with some confidence as arising from the changes in the stimuli. As a side-note, we stress that the auralizations used in this study were not notably quieter when there were high proportions of EVs, and the difference between versions with high and low EV proportions was relatively subtle. Any differences in rating were not attributable simply to overall changes in loudness.

Finally, to see whether certain components of the sound were particularly important for influencing people’s perceptions, participants heard a full-spectrum version of each auralization and also several filtered versions. If any of these filtered versions showed similar responses to the full-spectrum version, this might suggest that it is a subset of the frequency components in the full-spectrum version that was particularly contributing to people’s impressions. Ishiyama and Hashimoto [23] previously identified high-frequency sound (which they defined as >1000 Hz) as particularly contributing to annoyance from traffic noise.

## Method

### Participants

Data were gathered from 31 participants (8 male) with a mean age of 24 (range 19 to 58, SD = 9.79). The participants were recruited opportunistically from the staff and students of a UK university and received no payment for taking part. Eight participants reported having lived near a major road at some point in their lives. Participant testing was approved by the University of Bath Psychology Ethics committee with reference 13–169.

### Materials and design

Recordings of vehicle noise from earlier vehicle pass-by tests were used to create the auralizations for this study. Full details can be found in Kennedy, Rice and Walker (in prep), and to repeat the whole process here in full would introduce an unnecessary level of complexity. But in brief, the recordings were processed through the following procedure to generate the auralizations. First, the pass-by test recordings (from a conventional Renault Twingo and an electric Citröen C0) were de-Dopplerised and corrected for attenuation effects before being truncated to a region relatively close to the pass-by microphone–approximately +/- 25 m. A simple model of directivity was achieved by splitting the test data into sections before and after the pass-by point, and source spectra were calculated for the two regions. These source spectra were used to generate mutually uncorrelated source signals, which were processed to apply Doppler and attenuation effects equivalent to a vehicle travelling on a stretch of national road. Finally, so that the scenes correctly simulated the experience of a human listener, the signals were auralized using Head Related Impulse Responses (HRIRs) so that the sound over headphones contained similar head-related information as would be experienced by a listener in open-field conditions. The high spatial resolution HRIRs came from the public-domain CIPIC database measured at the U.C. Davis CIPIC Interface Laboratory, which includes extensive measurements of the standard KEMAR binaural head to generate a general HRIR database.

The auralizations simulated a basic 4-lane roadway, with vehicles moving from left to right in the lanes closest to the point of reception and from right to left in the two lanes furthest from the point of reception. The road traffic environment was generated so that 10 vehicles were placed in each lane, with a random gap between each vehicle of between 1.7 and 2.3 s. The simulated road environment was 250 m long with the listener placed at the halfway point. The sound files were generated beginning when two vehicles had already passed the listener in the near lane (meaning each recording began with the sound of vehicles receding) and ended when there were still two vehicles yet to pass the point of reception (meaning each ended with the sound of vehicles approaching). This produced the listening experience a constant steady traffic flow. The sound files were approximately 34 seconds in length and copies of the auralizations can be downloaded from https://osf.io/m6yh4/, where copies of the experimental data can also be found.

Six versions of the auralization were produced, each with a different mixture of conventional and electric vehicles: 0%, 20%, 40%, 60%, 80% and 100% EV. As well as the original full-spectrum recording, each auralization was also filtered to give a 0–100 Hz lowpass filtered version, a 100–500 Hz bandpass filtered version, and 500–2000 Hz bandpass filtered version, and a highpass filtered version retaining only frequencies over 2000 Hz. As such, there was a total of 6 traffic mixtures × 5 filters = 30 conditions.

The procedure was controlled by a laptop computer and participants listened to the sounds using Superlux HD-681 Evo headphones, which the experimenter ensured were worn on the correct ears. The computer’s volume was set at an appropriate, realistic and non-painful level before the study and remained the same for every participant.

The study design was entirely repeated-measures: every participant judged every condition, meaning the conditions’ ratings are directly comparable to one another.

### Procedure

Participants were tested individually in a quiet room. The procedure lasted around 25 minutes.

**Fig 1:**
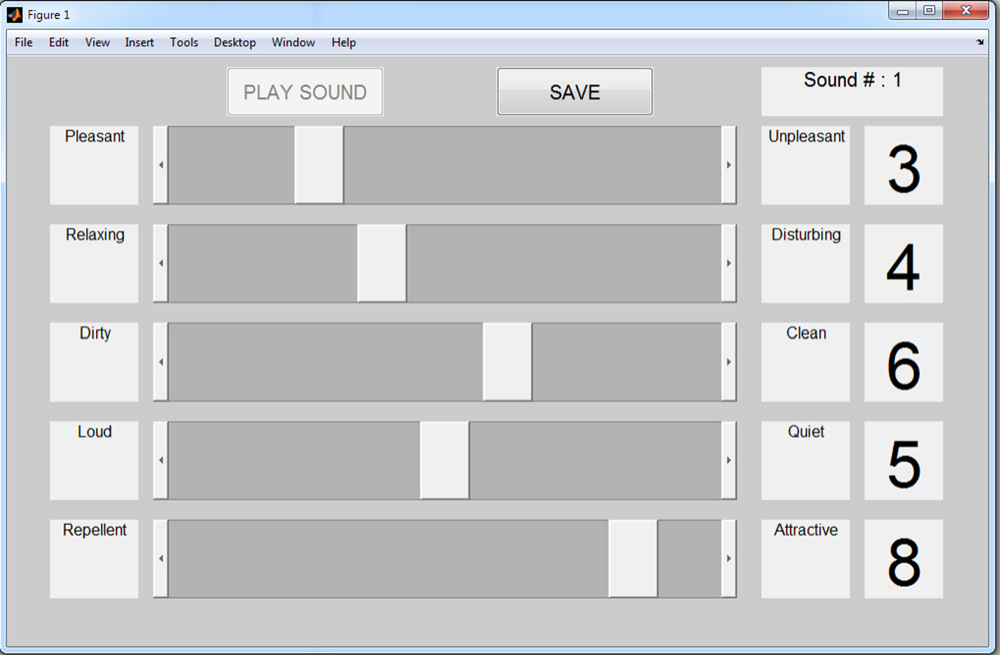
A representative screenshot of the test software in use.

After participants gave written consent and basic demographic information, custom software presented them with the 30 auralizations in random order. Participants began each trial by pressing a button on the computer screen, at which point the next auralization would start to play over the headphones. As they listened, participants could adjust five on-screen sliders to rate the sound from 1 to 9 on five scales (see Fig 1 for a screenshot). The scales were labelled with the following end-point labels: pleasant-unpleasant, relaxing-disturbing, dirty-clean, loud-quiet, repellent-attractive (after testing, the first two scales had their scores reversed so that a high score represented a more positive rating on all the scales). As the sliders were adjusted, a number at the end of the scale told participants which integer score, on the scale 1 to 9, they were registering. Sliders could continue to be adjusted once the sound had ended. Once participants were satisfied with the five ratings they had given to an auralization, they pressed a button to submit these scores and begin the next trial.

## Results

**Fig 2:**
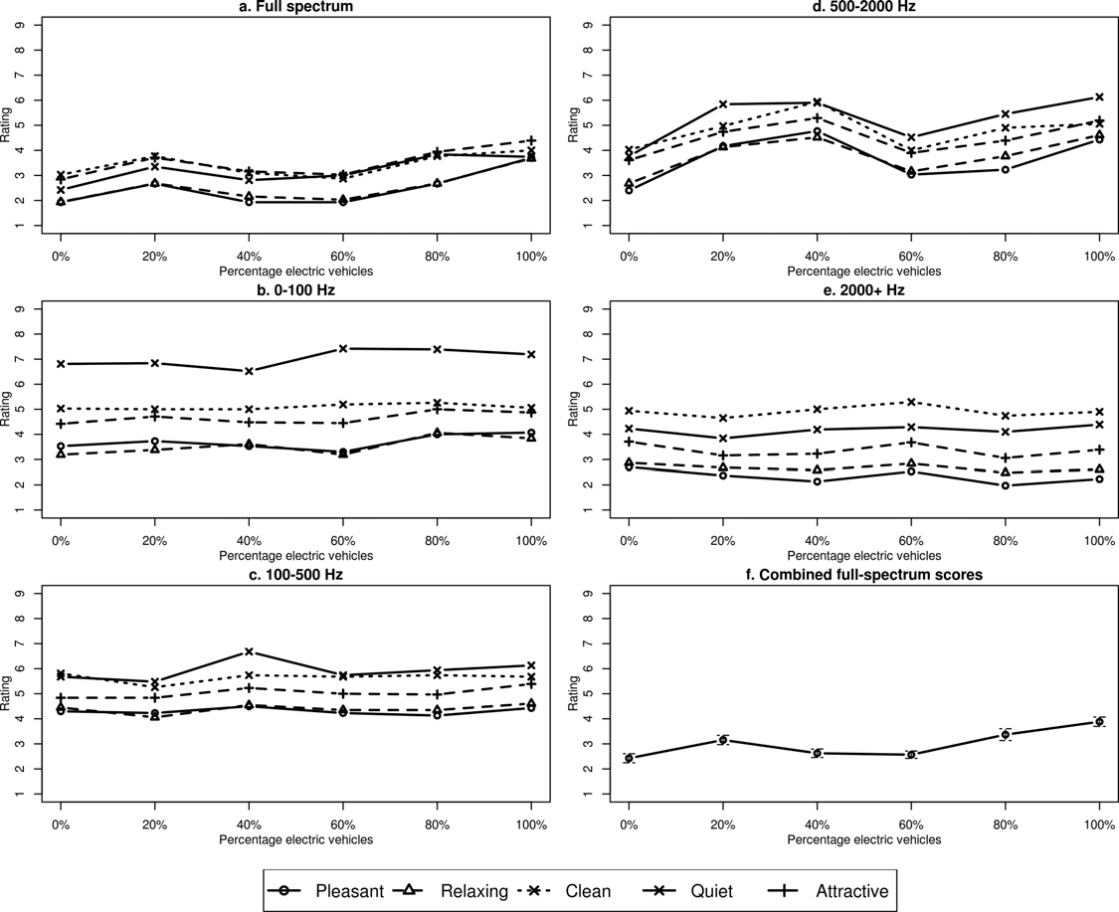
Subjective ratings for full-spectrum (a) and filtered (b-e) auralizations, plus combined preference ratings for the full-spectrum sounds (f), as a function of percentage of EVs in the auralization. Legend applies to a-e only; error bars in (f) represent standard errors of the mean

Fig 2A shows mean ratings across the participants for the full-spectrum auralizations, as a function of the percentage of EVs. Each line on the graph represents one of the five perceptual dimensions that the participants rated. Fig 2B–2E show the same information for the four filtered versions of the traffic sounds and Fig 2F shows the full-spectrum ratings from Fig 2A but with the five perceptual dimensions collapsed together to produce overall ratings of subjective experience.

A few observations can be made from looking at Fig 2. First, the 80% and 100% EV scores, on right-hand side of the full-spectrum data (Fig 2A and, particularly, Fig 2F), are generally higher than the points on the left-hand side of the graph, suggesting ratings are more positive with higher proportions of EVs in the auditory scene. The exception to this pattern is the 20% EV condition, where ratings for some reason are higher than in the 0%, 40% and 60% conditions.

Second, it is notable that the ‘pleasant’ scores (circles) and ‘relaxing’ scores (triangles) are highly correlated across Fig 2A–2E, suggesting these two dimensions were seen as closely linked by the participants (correlations between these two measures ranged from *r* = .46 to *r* = .92 across the 30 conditions, with a mean of *r* = .73).

Finally, it is striking how the ‘quiet’ rating is much higher in the 0–100 Hz filtered auralizations than in any other version, suggesting that energy in this frequency band does not much contribute to people’s perceptions of how loud traffic feels. This effect is presumably related to the human ear’s relatively poor sensitivity to low-frequency sounds [25].

The data in Fig 2A were reduced to focus on our specific research questions. After the first two perceptual scales had their scores reversed, all five scales worked in the same way such that a higher score meant a more positive evaluation of the auralization. We could therefore average the pleasant, relaxing, clean, quiet and attractive ratings to get a single combined ‘preference rating’ measure. Collapsing the ratings in this way was justified because the five measures showed strong consistency: Chronbach alpha measures ranged from .70 to .91 across the 30 conditions, with a mean of .82. The collapsed data are shown in Fig 2F, and Table 1 provides Holm-Bonferroni-corrected *t*-test results showing which conditions from this graph were rated as significantly different from one another. The bottom two rows of Table 1 suggest that participants preferred mixes of 80% and 100% EV over auralizations with less than 80% EV. This suggests there might be a critical point between 60% and 80% EV at which the experience of hearing this traffic noise changes almost qualitatively. The one datum that does not fit this pattern is the 20% EV condition, which was rated significantly higher than the 0%, 40% and 60% EV conditions. Whilst significantly lower than the 100% EV rating, the 20% EV rating was not significantly different from the 80% EV rating. As discussed below, we believe this is most likely an experimental artefact and the higher rating for the 20% condition should be ignored.

**Table 1:** Corrected *t*-test *p*-values for pairwise comparisons of preference rating scores from Fig 1F.

	0% EV	20% EV	40% EV	60% EV	80% EV
20% EV	**< .001**				
40% EV	.32	**.019**			
60% EV	.71	**.005**	.71		
80% EV	**< .001**	.71	**.006**	**.006**	
100% EV	**< .001**	**.006**	**< .001**	**< .001**	.13

**Fig 3:**
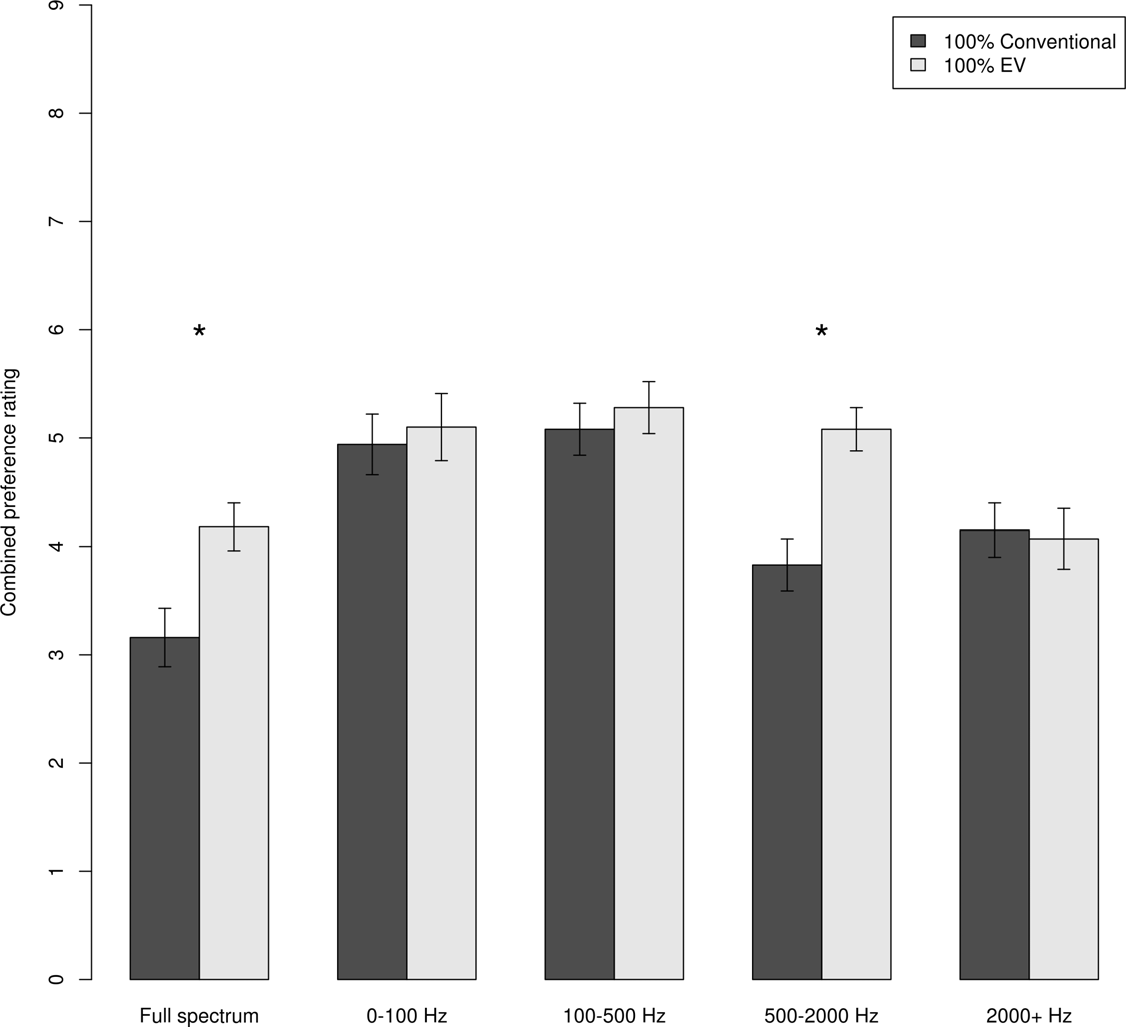
Mean preference ratings for simulated 100% conventional and 100% electric vehicle traffic noise. The five pairs show the five filtering conditions. Error bars represent standard errors of the mean

Finally, to address the possible real-world impact of EV adoption on listeners even more clearly, we focused on the most straightforward case: those auralizations with 100% conventional vehicles versus those with 100% electric vehicles. These data are shown in Fig 3, which presents, for each filtering condition, the mean overall preference rating for the 100% conventional and the 100% EV auralizations. Fig 3 shows that participants preferred 100% EV auralizations over 100% conventional versions when listening to the full-spectrum recordings. Moreover, Fig 3 suggests that it is information in the 500–2000 Hz frequency band that might underpin this preference, as the data in this condition show the same pattern as the full-spectrum version, whereas all the other filtered versions show no difference between the 100% conventional and 100% EV conditions. A two-way repeated-measures analysis of variance showed a significant main effect of Filtering Condition (Wilks’s λ = .16, *F*(4,27) = 36.73, *p* < .001) and a main effect of EV Mix (Wilks’s λ = .20, *F*(1,30) = 119.63, *p* < .001). The Filtering Condition × EV Mix interaction was, as would be expected from Fig 3, also significant (Wilks’s λ = .32, *F*(4,27) = 14.43, *p* < .001). Holm-Bonferroni-corrected pairwise *t*-test comparisons confirmed that the 100% conventional and 100% EV conditions were significantly different for the full spectrum (*p* < .001) and the 500–2000 Hz (*p* < .001) conditions, but not for the 0–100 Hz (*p* = .42), 100–500 Hz (*p* = 1.00) or 2000+ Hz (*p* = 1.00) conditions.

## Discussion

As we consider the electrification of the world’s vehicle fleet, it is important to think about the impact this development is likely to have on people, particularly given evidence on the detrimental effects of traffic noise today. This study provides what we believe is the first clear data to estimate the possible impact of qualitatively changed vehicle noise on people living near national roads as the vehicle fleet becomes increasingly electrified. The data suggest that, thanks to different engine tones, EV growth is likely to make current negative perceptions of road noise less bad. This choice of words is deliberate: ratings in this study were on a 9-point scale, which means any rating below 5 is a negative rating. As Fig 3 shows, even the highest ratings seen here were only around this midpoint, suggesting the best we can hope for might be a shift from negative perceptions to a more ambivalent experience. Although an increase in EV numbers looks like it should be a good thing in terms of noise exposure, it is unlikely anybody will actually start to *like* the sound of a busy road.

On the other hand, in the longer term, the shift from conventional engines to EVs might plausibly produce even more substantial changes in perceptions than described here. Our analysis examined a situation in which engine sound changed but other sources of noise–particularly the vehicle’s tyres–remained essentially the same as for conventional vehicles. However, the mass of an electric engine is substantially lower than a conventional engine, which reduces overall vehicle mass considerably. As such, future EVs might not require tyres with the same width and adhesive properties as a conventional vehicle. This means that in the longer term, a shift to EVs should reduce tyre noise as well as change the tone of engine noise–meaning the effect presented in this study is probably a conservative estimate of the improvements EVs offer.

Another insight from this study is that negative perceptions of vehicle noise seem to be particularly tied to energy in the 500–2000 Hz band. Ishiyama and Hashimoto [23] previously implicated ‘high-frequency’ sound components in causing traffic noise annoyance, with high-frequency defined as >1000 Hz. Our findings largely support theirs, and extend their work in two ways. First, our study shows the effect is also present with a more sensitive, multidimensional measurement of experience, rather than the simple five-point annoyance scale they used. Second, by showing that frequency components over 2000 Hz do not contribute to negative perceptions, our study places an upper-limit on Ishiyama and Hashimoto’s definition of ‘high-frequency’. Now this 500–2000 Hz band is more clearly established, vehicle and highway engineers might usefully improve the experience of people living near roads if they pay attention to reducing energy in this zone–an approach which should work even for conventional vehicles.

The methods used here have certain strengths. Not only did our laboratory approach provide considerable experimental control, but the method in which each judgement was made for a single auralization in isolation meant the procedure should not show biases that might arise in a two-alternative forced-choice procedure when, for example, people cannot tell two versions apart. On the other hand, it is possible that, although used in previous research [21], conscious, immediate perceptual ratings do not capture the full experience of being exposed to traffic noise from major roads; there might be unconscious responses or long-term adaptations to the noise that our methods do not address. Future studies might usefully include longer-term exposure and supplement conscious ratings with physiological measures (e.g. heart-rate variability) and hormonal measures (e.g., cortisol levels) to assess more implicit responses.

On a related note, we must note that no experimental research could ever hope to simulate the experience of gradual changes in the proportion of EVs in the vehicle fleet over many years. It remains possible that if a country moved to a high proportion of EVs over a period of decades, people living or working near national roads would not notice the slow changes in the way they apparently did in this study. However, that does not negate the main finding here, which is that growth in EVs is unlikely to have a negative effect on people through noise mechanisms. If EVs were preferred in this immediate comparison, there is no reason to suspect they would not be preferred after longer-term change.

Finally, one datum in our results that did not fit the overall pattern was the preference for 20% EV auralizations over 0%, 40% and 60% versions. With hindsight, we now believe this to be an experimental artefact that should not be interpreted to mean anything. The auralizations that included a mixture of conventional vehicles and EV had the vehicles appear in the same random order for each participant. It appears that the auralization for the 20% condition, by an accident of randomization, had a particularly high number of EVs near the beginning of the sound file. This meant a participant’s first impressions of this auralization would have mostly been shaped by EVs.

## Conclusions

People’s subjective ratings of simulated traffic noise from a major road were improved (in the sense that they became less negative) as the proportion of electric vehicles increased–particularly once the mix reached over 80% electric vehicles. This suggests electrification of the vehicle fleet is unlikely to have a detrimental effect on people living near such roads through changes in noise. Moreover, negative perceptions of traffic noise seemed particularly tied to energy in the 500–2000 Hz range, meaning the experiences of people living near major roads might usefully be improved by engineering interventions that specifically target noise in this band, even for conventional vehicles.
